# Penetration depth of cold atmospheric plasma into biological tissue: a review

**DOI:** 10.3389/fbioe.2026.1764941

**Published:** 2026-02-16

**Authors:** Dong Jiang, Jiashuo Zhang, Zhixin Liu, Yilin Yu, Li Xiao, Mi Ai, Ming Luo, Ollie Yiru Yu, Yingguang Cao, Ke Song

**Affiliations:** 1 Department of Stomatology, Tongji Hospital, Tongji Medical College, Huazhong University of Science and Technology, Wuhan, China; 2 School of Stomatology, Tongji Medical College, Huazhong University of Science and Technology, Wuhan, China; 3 Hubei Province Key Laboratory of Oral and Maxillofacial Development and Regeneration, Wuhan, China; 4 Faculty of Dentistry, The University of Hongkong, Hong Kong SAR, China

**Keywords:** biological tissue, cold atmospheric plasma, histological layers, penetration depth, reactive oxygen and nitrogen species, tissue model

## Abstract

Cold atmospheric plasma (CAP) is a non-thermal plasma generated near room temperature that has broad medical applications in the medical field, including antitumor, antimicrobial, and anti-inflammatory effects, promotion of tissue regeneration, and enhancement of transdermal and mucosal drug delivery. However, there is currently a lack of standardization regarding the indications for CAP and its application parameters, resulting in varying degrees of histological penetration depths reported in different studies. Therefore, to further promote the safe and effective clinical application of CAP, the histological levels at which CAP can be applied must be clearly defined. Here, we review the depth of tissue penetration achieved by CAP under various conditions and analyze the key factors influencing penetration depth, using this knowledge to propose how these factors should be adjusted for different application requirements to achieve safer and more precise therapies.

## 1 Introduction

### 1.1 What is plasma?

Plasma is the fourth state of matter and is distinct from solids, liquids, and gases (Hoffmann et al., 2013). It is an ionized gas composed of electrons, ions, free radicals, and excited particles, forming a mixture that is electrically neutral overall. Plasmas can be classified by their thermodynamic equilibrium into two broad categories, namely high-temperature (fully ionized) and low-temperature plasmas (partially ionized). High-temperature plasma is fully ionized, in which all particle species are at the same temperature, resulting in extremely high gas temperatures. Low-temperature plasmas are not fully ionized and can be further divided into thermal (also known as equilibrium plasmas) and non-thermal plasmas (also known as non-equilibrium plasma or cold plasma). Cold atmospheric plasma (CAP) is a cold plasma in which the electron temperature is high, whereas the temperatures of the other species remain near room temperature; therefore, the overall temperature of CAP is close to room temperature.

### 1.2 What can plasma do?

CAP contains numerous active components, including reactive oxygen species RONS molecules, electric fields, and ultraviolet (UV) radiation (Chauvin et al., 2017; Liu et al., 2015). With these active components, CAP can exert sterilizing and anti-inflammatory effects, promote healing, exhibit antitumor activity, and perform a series of functions in the field of oral medicine (Duarte and Panariello, 2020; Yan et al., 2015; van Gils et al., 2013).

In terms of sterilization, those RONS molecules combined with UV radiation and electric fields can strongly eliminate a large number of bacteria, even the multidrug-resistant bacteria (Boekema et al., 2021). When addressing biofilms, CAP not only eliminates bacteria within the biofilm but also physically disrupts and detaches the biofilm, ensuring that bacteria deep within are also targeted (Schmidt et al., 2019).

In controlling inflammation and promoting wound healing, CAP (low to moderate doses) can not only achieve the aforementioned sterilization but also promotes angiogenesis, improves microcirculation, enhances cell proliferation and migration, and modulates inflammatory responses (Zhang et al., 2025; Rajić et al., 2025; Nicol et al., 2020; Tornín et al., 2023).

Moreover, the selective killing effect of CAP on tumor cells represents a groundbreaking discovery. CAP can induce apoptosis, necrosis, and other forms of programmed cell death in tumor cells, and can also cause cell cycle arrest. Numerous studies have found that combining CAP with other antitumor therapies can achieve better outcomes (VONW et al., 2019; Faramarzi et al., 2021; Peng et al., 2024; Nitsch et al., 2024; Perrotti et al., 2022; Gherardi et al., 2018).

In the field of dentistry, CAP can be used for root canal disinfection, treating periodontal disease and oral mucosal disorders, removing oral biofilms, as well as for teeth whitening and implant surface modification (Shi et al., 2015; Yao et al., 2021; Negrescu et al., 2024; Sung et al., 2013). Compared to traditional medications, CAP can better penetrate into narrow spaces such as root canals and gingival sulci. Beyond the aforementioned fields, the application scope of CAP continues to expand. Emerging application areas include medical device sterilization (Kramer et al., 2022; Fridman et al., 2006), promoting blood coagulation (Ke and Huang, 2016; Guo et al., 2018), virus inactivation (Xia et al., 2019; Bakhtiary et al., 2025), facilitating tooth remineralization (Nie et al., 2018a) and so on.

## 2 Factors influencing the penetration depth of active ingredients generated by CAP within tissue

**FIGURE 1:**
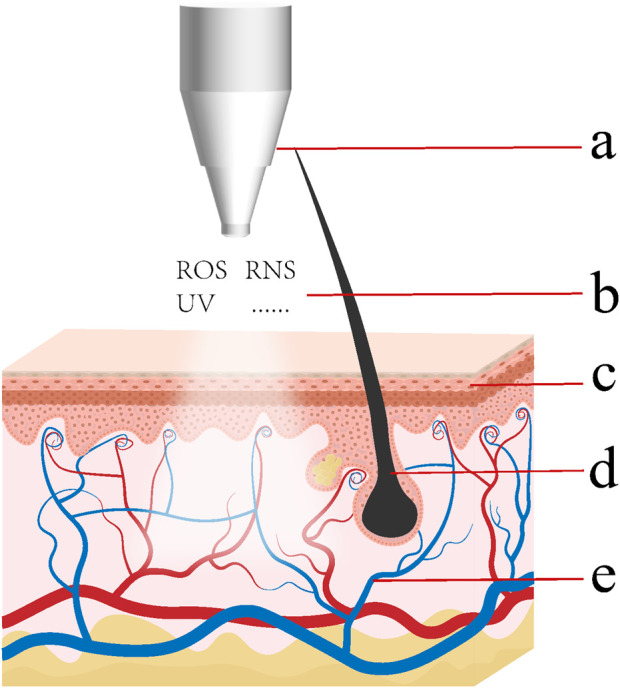
Factors that can influence the penetration depth of active ingredients generated by CAP within tissue. **(a)** The type and parameters of CAP devices and the type of working gas can influence the variety quantity of active ingredients contained within the CAP. **(b)** The manner of reaction (direct or indirect), the distance and duration can influence the types and quantities of active species delivered to tissues. Gas flow can propel the movement of reactive species. **(c)** Tissue structures and components can affect the barrier effects, the ability of conveying active species, generating secondary RONS and initiating intercellular signaling processes. **(d)** Hair follicles or supplementary hollow microneedles can provide a rapid penetration pathway for active substances. **(e)** Metabolic Activity and Immune Regulation can mediate long-distance and long-term effects.

The depth of CAP’s effects on the tissue is influenced by numerous factors. The primary active components generated by CAP are ROS and RNS (collectively referred to as reactive oxygen and nitrogen species, RONS). The types and amounts of RONS delivered, particle entrainment by gas flow, tissue barrier properties, secondary RONS generation, tissue metabolism, cell–cell interactions, and transport by interstitial fluid and blood can all influence the penetration depth (Figure 1).

### 2.1 Physical and chemical traits of CAP

#### 2.1.1 Traits of different reactive components

CAP is rich in reactive species, including reactive oxygen species (ROS), reactive nitrogen species (RNS), charged particles, excited state atoms and molecules, electric fields, and UV radiation. The active species in CAP can initiate a cascade of chain reactions upon contact with a substrate, generating diverse reactive species, including long-lived species such as O_3_, H_2_O_2_, NO_2_^−^, and NO_3_^−^, as well as short-lived species such as O, OH, and NO. Different types of reactive species exhibit distinct permeation abilities. Generally, long-lived species penetrate to greater depths than short-lived ones. Moreover, various physicochemical properties, such as solubility, molecular size, and reactivity, also influence the penetration depth of reactive species (Verlackt et al., 2018; Gelker et al., 2020). Table 1 contains some common short-lived species and long-lived species as well as their main biomedical effect. Some factors, including the parameters of the CAP device, the type of working gas, and whether the application is direct or indirect, can all influence the types of reactive species.

**TABLE 1:** Characteristics and biological effects of commonly encountered reactive species in CAP.

Lifespan	Species	Main biomedical effects
Short-lived species	·OH	Low doses: Enhances angiogenesis and tissue regeneration, Promote cell proliferation, migration, and wound healing High doses: Strong oxidizer; damages bacterial membranes, DNA, and lipids; induces apoptosis in cancer cells
	^1^O_2_	Low doses: Promotes cell proliferation, wound healing, and angiogenesis via redox signal transmission High doses: Induces oxidative damage, killing pathogens or cancer cells
	·O_2_^−^	Low doses: Activates pro-survival pathways (e.g., NF-κB) and immune responses High doses: Causes oxidative stress, DNA damage, and inflammation
	ONOO^−^	Low doses: Regulates redox-sensitive signaling pathways, potentially promoting cell proliferation and tissue repair in normal cells High doses: Induces severe oxidative stress, causes nitrative damage to cellular proteins
Long-lived species	H_2_O_2_	Low doses: Promotes wound healing by enhancing cell proliferation, migration, and angiogenesis. Exerts antimicrobial effects. Modulates inflammation by reducing pro-inflammatory cytokines and promoting tissue repair factors High doses: Oxidates DNA, lipids and proteins
	O_3_(Ozone)	Low doses: Promotes wound decontamination, generate ·OH via secondary pathways High doses: Oxidates DNA, lipids and proteins strongly
	NO_2_^−^/NO_3_^−^	Generating secondary species (primarily ONOO^−^)
	NO	Low doses: Promotes wound healing and angiogenesis. Exerts antimicrobial effects against bacteria and fungi through membrane disruption and metabolic interference. Modulates anti-inflammatory responses by reducing pro-inflammatory cytokines High doses: Killing pathogens, cancer cells or even healthy cells

Here, the distinction between “low dose” and “high dose” is primarily based on the biological effects they induce rather than a fixed numerical threshold. Low dose typically refers to the dose range at which plasma treatment produces beneficial or protective biological effects under specific parameter combinations. High dose generally denotes the dose range that induces inhibitory or destructive biological effects. Therefore, researches often require determining thresholds for specific scenarios through dose-response curves. The short-lived reactive species usually have lifetimes on the order of microseconds or shorter and long-lived species can persist on the order of seconds to hours.

#### 2.1.2 Type and parameters of CAP devices

**FIGURE 2:**
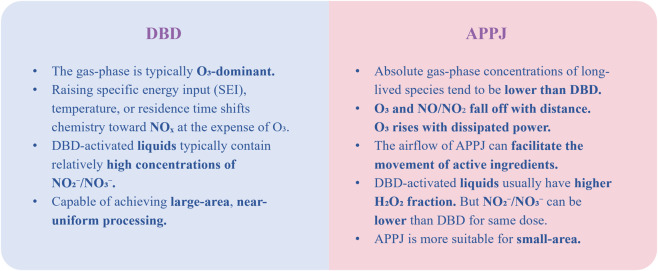
Comparison of DBD and APPJ.

The commonly used low-temperature plasma devices mainly include atmospheric pressure plasma jet (APPJ) and dielectric barrier discharge (DBD). Figure 2 illustrates the main differences between DBD and APPJ. There is also a modified form of DBD, namely the floating-electrode DBD (FE-DBD), in which biological tissue can serve as part of the grounded electrode. Increasing the applied power can enhance the penetration depth of CAP within a defined range (Gelker et al., 2018; Gelker et al., 2019). In addition, the power supply frequency can also significantly affect the penetration behavior. Some studies have reported that microsecond-pulsed DBD exhibits stronger penetration than nanosecond-pulsed DBD (Gelker et al., 2018; Gelker et al., 2019).

#### 2.1.3 Type of working gas and gas flow rate

For DBD, the working gas is mainly air, whereas APPJ commonly uses inert gases, reactive gases, or gas mixtures. When pure inert gases (typically Ar or He) are used as the working gas of APPJ, the resulting effects are primarily physical, with limited radical generation and shallow penetration depth. Moreover, studies have demonstrated that under identical discharge parameters, Ar-based APPJ can generate more reactive species than He-based APPJ and is more effective in disrupting intercellular E-cadherin, thereby enhancing the permeability of the epidermis (Lee et al., 2018). However, when reactive gases or inert–reactive gas mixtures are used, the specific gas type can significantly influence the types of reactive species generated, which in turn results in markedly different penetration depths. In terms of the gas flow rate, Szili et al. reported that when in the absence of gas flow, the penetration of reactive species in deionized water treated with He plasma decreased. Therefore, it can be considered that gas flow can influence the penetration of reactive species (Szili et al., 2015).

### 2.2 Treatment modality

#### 2.2.1 Direct or indirect treatment

Direct treatment refers to CAP being applied in direct contact with tissues, where all reactive components generated by the plasma (including charged particles, short-lived and long-lived species, UV radiation, electric fields, and heat) act simultaneously on the target cells or tissues (Malyavko et al., 2020).

**FIGURE 3:**
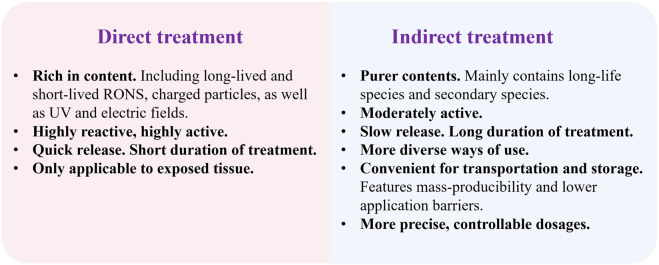
Comparison of direct treatment and indirect treatment.

Indirect treatment refers to CAP first being used to activate a liquid medium, producing a plasma-activated medium (PAM) enriched with long-lived reactive species. PAM is subsequently applied to the target tissues (Dai et al., 2023). Since the primary components in PAM are long-lived species, PAM may exhibit stronger permeability and exert its effects for a longer duration than direct treatment, a finding also confirmed in the study by Liu et al. (Liu X. et al., 2018). Another unique advantage of PAM is that it can be delivered (usually injected) into deep tissues, thereby exerting its effects rapidly *in vivo*. Therefore, PAM is considered a promising new therapeutic approach for treating various diseases like tumors within the body. Numerous studies have already applied PAM in mechanism research and animal experiments, confirming the efficacy of this treatment method (Nakamura et al., 2017; Yao et al., 2025; Jo et al., 2022; Takeda et al., 2017; Cheng et al., 2020; Saadati et al., 2018). Figure 3 illustrates the main differences between direct treatment and indirect treatment of CAP.

#### 2.2.2 Distance and duration of action

In a study in which a gelatin model was used as the target of CAP treatment, it was found that within a certain range, the penetration depth increased monotonically with the treatment time and decreased with the distance from the plasma source to the tissue surface (Wang et al., 2024a; von Woedtke et al., 2020). Notably, excessively long treatment durations or excessively short treatment distances may result in tissue dehydration and damage.

### 2.3 Tissue characteristics

#### 2.3.1 Tissue structures

Several studies have demonstrated that dense tissues like the stratum corneum have a significant barrier effect on the penetration of reactive components. Although previous studies have confirmed that CAP can temporarily weaken the barrier properties of the stratum corneum through mechanisms such as lipid peroxidation and electroporation, CAP permeability in intact skin remains substantially lower than that in skin from which the stratum corneum has been removed (Liu X. et al., 2018).

#### 2.3.2 Tissue components

Tissue components can affect the efficiency of active substance uptake as well as their subsequent penetration into deeper layers of the tissue. In the cell membrane, aquaporins (AQPs) facilitate the entry of hydrophilic RONS into cells (Yusupov et al., 2019; Bogaerts et al., 2019). In contrast, cholesterol inhibits oxidation and pore formation, thereby maintaining membrane stability and limiting RONS entry (Chen et al., 2014). For instance, cancer cells typically exhibit elevated AQP expression and reduced cholesterol in their membranes, which facilitates the entry of RONS into these cells. The extent of CAP effects in tissues is not necessarily directly correlated with the capacity of the entry of RONS into cells (Svarnas et al., 2017), but is instead influenced by a complex interplay of multiple factors. In some instances, a substantial uptake of RONS by superficial cells may reduce their availability for diffusion into deeper layers. Conversely, under other conditions, the entry of RONS into superficial cells may initiate intercellular signaling processes that propagate biological effects into deeper tissue regions. In addition, factors such as the content and fluidity of tissue fluid, the number and functional status of mitochondria within cells can also influence the depth of CAP effects in tissues (von Woedtke et al., 2020; Chen et al., 2014; Zorov et al., 2014; Dan Dunn et al., 2015).

#### 2.3.3 Systemic effects, metabolic activity, and immune regulation

When tissue metabolism is highly active, the extracellular matrix exhibits increased hydration and fluidity, which facilitates the diffusion of RONS generated by plasma. Moreover, CAP can activate redox signaling pathways and modulate metabolism-related immune responses, thereby more readily eliciting systemic effects in metabolically active tissues. Some researchers have argued that the direct impact of plasma on tissues is confined to superficial layers and generally persists for only a few minutes, suggesting that more durable and deeper effects depend on host metabolism and immune mechanisms (Graves, 2014). Mizuno et al. demonstrated that in mice bearing multiple tumors, CAP treatment of a single tumor significantly suppressed the growth of distant, untreated tumors, which supports the above concept (Mizuno et al., 2017). This finding highlights the potential of CAP in inducing systemic immune effects, underscoring the pivotal role of metabolic and immunological interplay in mediating its therapeutic efficacy.

## 3 How to regulate the effective depth of CAP in tissues

After explaining the factors that influence the effects of the penetration depth of CAP on tissues, we can explore how the penetration depth can be regulated.

### 3.1 Adjust the parameters of the plasma generator, working gas, irradiation distance, and duration

As noted above, one can choose to add small amount of oxygen or nitrogen to the inert gas as the working gas, appropriately increase the power of the plasma generator, decrease the distance between the plasma source and the tissue, and extend the exposure time under the premise of ensuring biosafety when seeking to enhance the effective depth.

### 3.2 Applying an additional electric field

It has been reported that the application of a weak electric field (<20 V/cm) to hydrogels can enhance the permeability of NO_2_^−^ and H_2_O_2_, irrespective of the field orientation relative to the direction of particle penetration (He et al., 2016). Don’t forget to ensure the biosafety.

### 3.3 Moderate increase in water content

Kim et al. reported that in an artificial wound model infected with bacteria, covering the wound surface with a thin layer of PBS buffer enhanced the sterilization effect of CAP on biofilms (Chen et al., 2020). This may be attributed to the generation of more reactive species in the liquid phase plasma, as well as the roles of bubble-mediated transport and capillary action.

### 3.4 Use microneedles

The combination of CAP with appropriately selected types and sizes of microneedles can significantly enhance the penetration depth of reactive species in tissues. A previous study found that the use of a hollow-structured microneedle patch can significantly enhance the effects of CAP (Kos et al., 2017). However, another study suggested that conventional microneedles (which are withdrawn immediately after piercing the stratum corneum) do not significantly enhance the permeability of CAP (Nakamura et al., 2017). This may be due to the rapid closure of the pores owing to the elastic properties of the tissue. Therefore, the type and size of microneedles have a significant impact on whether CAP can effectively increase its penetration depth in tissues.

### 3.5 Flow of interstitial fluid

Approaches promoting the flow of interstitial fluid can also facilitate the delivery of reactive species into deeper tissue layers.

### 3.6 Delivering PAM to the required site is also an effective approach

PAM can be delivered directly (e.g., by injection) to any required site within the body, giving it a unique advantage in treating deep-seated lesions.

## 4 Safe operating range for plasma

Due to the diversity of existing CAP equipment, the controllability of its parameters, and the wide range of applications for CAP there is currently no standardized safety application specification. When focusing on the effects of CAP on living tissue, the parameters used in most researches are as follows: the voltage usually in the thousands of volts. To ensure the tissue temperature remains within a safe range (typically between 42 and 43 °C), the operating power of the CAP device generally ranges from several watts to tens of watts. Moreover, the typical energy density is less than tens of J/cm^2^. However, when applying CAP to fields such as sterilization and antitumor therapy, the goal is to induce localized tissue cell apoptosis or death. Therefore, the parameter range of CAP can be appropriately expanded.

When using CAP beyond safe dosage levels, large quantities of reactive species, heat, ultraviolet radiation and other substances can cause tissue damage. First, the excessive RONS can cause oxidative stress, thereby leading to lipid membrane peroxidation, DNA damage and protein carbonylation. At the same time, excessive doses of CAP can also cause tissue temperatures to rise excessively, leading to cell death. These factors can all lead to cell apoptosis or even necrosis, mitochondrial dysfunction and so on, resulting in clinically observable tissue damage alongside proliferative repair of surrounding tissues, pain or sensory abnormalities, and inflammatory responses. For instance, one study used FITC labelled dextran to indicate tissue damage within the mouse skin. They confirmed that CAP can cause direct damage to mouse skin and also found that 24–48 h after CAP exposure, the additional damage around the direct plasma damage was observed. This late damage was presented as oedema around the treated area, and was not subjected to initial direct plasma damage (Kos et al., 2017).

## 5 Methods for detecting the penetration depth of CAP in tissue models and tissues

Existing studies generally suggest that when CAP acts on tissues, RONS are the main active components. Consequently, most research has focused on measuring the penetration depth of RONS, and their associated biological effects. Some studies used tissue models or *in vitro* tissues as substitutes for living tissues. Therefore, in this review, the summary of CAP penetration depth will be organized according to different types of tissue models or living tissues.

### 5.1 Electron spin resonance (ESR) or electron paramagnetic resonance (EPR)

ESR is a magnetic resonance technique for detecting paramagnetic substances (those containing unpaired electrons) (Janzen and Blackburn, 1968; Suzen et al., 2017; Szili et al., 2018).

Conventional ESR is mainly used for detecting long-lived radicals, whereas spin-trapping ESR enables the detection of short-lived radicals by forming more stable spin adducts. Despite its high precision, this technique is limited by the biological toxicity or poor cell permeability of some spin traps, as well as the high cost of EPR, which sometimes restricts its application in biological tissues.

### 5.2 Colorimetric assay and UV–vis absorption spectroscopy

UV-Vis provides a simple and rapid method for detecting RONS. Certain species exhibit intrinsic UV absorbance, whereas others can be monitored through chromogenic probes that yield characteristic spectra upon reaction. Although sensitive and convenient, the method depends on probe specificity and may suffer from interference in complex biological samples. Below are some common chromogenic probes for detecting the penetration depth of CAP.

#### 5.2.1 o-Phenylenediamine (OPD) combined with horseradish peroxidase (HRP) can be used to detect H_2_O_2_

In the presence of H_2_O_2_, HRP catalyzes the oxidation of OPD, producing the yellow compound 2,3-diaminophenazine (DAP) (Hempen et al., 2005; Szili et al., 2014; Szili et al., 2017a).

#### 5.2.2 Indigo reagent detects ozone (O_3_)

Ozone (O_3_) oxidizes indigo dyes (e.g., indigo trisulfonate, indigo disulfonate sodium), resulting in the decolorization and the formation of colorless isatin derivatives.

#### 5.2.3 DPD (N,NDiethyl-p-Phenylenediamine)

The DPD colorimetric method is primarily used to measure chlorine levels; however, it measures any oxidants present. Some previous studies used DPD to detect O_3_ (Nie et al., 2018b).

#### 5.2.4 Griess reagent detects nitrite

Nitrite reacts with the Griess reagent to form an Azo dye with a maximum absorption wavelength at 540 nm (Liu et al., 2015; Nie et al., 2018a; He et al., 2016; He et al., 2017; Zhang et al., 2019).

#### 5.2.5 KI-starch reagent detects ROS

The KI–starch reagent serves as a universal ROS detector that can detect several ROSs with oxidation potentials >0.54 V (Kawasaki et al., 2020; Liu D. et al., 2018; Kawasaki et al., 2016; Kawasaki et al., 2019; Ghimire et al., 2019).

#### 5.2.6 Other chromogenic probes

An increasing number of Colorimetric Assay are currently being developed, though they may not yet have been applied to CAP’s detection of biological tissue activity. For instance, a research reported a novel colorimetric and near-infrared fluorescent probe (pyridin-4-ylmethyl (Z)-2-cyano-2-(3-((E)-4-hydroxystyryl)-5,5-dimethylcyclohex-2-en-1-ylidene)acetate diphenyl phosphinate group (AN-DP)) based on isophorone and phosphinate groups for ONOO^−^detection (Gu et al., 2020).

### 5.3 Electrochemical methods

The electrochemical methods utilize electrochemical sensors to selectively detect different RONS based on their redox potential differences by adjusting the working potential. Various electrochemical and biosensors have been developed for different RONS (Malf et al., 2019; Deshpande et al., 2021; Hu et al., 2020; Li et al., 2020; Taheri et al., 2024; Xu et al., 2018). Although these techniques offer high sensitivity, rapid response, and the potential for miniaturization, their limitations include cross-interference among RONS species and disturbances from other substances in biological matrices and environments (Saeidi et al., 2023).

### 5.4 Fluorescent probe method

Fluorescence-based visualization has been continuously innovated in recent years, and fluorescent probes are now widely used for detecting RONS in tissues. They provide high sensitivity, strong selectivity, low invasiveness, and good biocompatibility, and can be targeted to subcellular organelles. Moreover, they can be combined with confocal microscopy or two-photon imaging to enable real-time observation of the spatiotemporal distribution of RONS in live cells and tissues. Below are some common fluorescent probes.

#### 5.4.1 2′,7′-Dichlorodihydrofluorescein diacetate (DCFH-DA) probe and its analogues detect ROS

DCFH-DA and its analogues are commonly used for detecting RONS within cells. However, their signals may be affected by interference from other cellular components.

#### 5.4.2 Amplex^®^ red reagent (10-Acetyl-3,7-Dihydroxyphenoxazine)Detects H_2_O_2_

Amplex® Red is a sensitive probe for H_2_O_2_ and peroxidases, producing red fluorescent resorufin upon reaction (Liu et al., 2015; He et al., 2016; Kim et al., 2011; Zhang et al., 2019; Dobrynin et al., 2012).

#### 5.4.3 5(6)-Carboxyfluorescein (CF)

When assessing the penetration depth of CAP into hydrogels, CF can be encapsulated at high concentration in vesicles where it is self-quenched. Because CAP causes vesicle rupture, CF is diluted, and quenching is relieved, resulting in enhanced fluorescence (Szili et al., 2017a; Marshall et al., 2013).

#### 5.4.4 Dihydroethidium (DHE) detects superoxide anion

DHE probe provides high sensitivity and enables visualization of intracellular superoxide generation (Bernhardt et al., 2019).

#### 5.4.5 Other fluorescent probes

Other fluorescent probes can also detect RONS; however, they have not yet been applied to assess the effects of CAP on tissue models or tissues. These include dihydrorhodamine 123, indigo green, 1,3-diphenylisobenzofuran, Azulene-Derived Fluorescent Probe (Murfin et al., 2019) and so on.

### 5.5 Chemiluminescence assay

#### 5.5.1 Lucigenin (*N*-Methyl-Acridinium Nitrate) detects superoxide anion

Lucigenin is a chemiluminescent probe commonly used to detect superoxide. Lucigenin is membrane-impermeable and therefore detects extracellular ROS only (Caldefie-Chézet et al., 2002).

#### 5.5.2 Luminol(3-Aminophthalhydrazide)detects peroxide

Luminol can be oxidized by various ROS in the presence of catalysts to produce chemiluminescence. Typical catalysts include multivalent metal ions and peroxidase enzymes such as horseradish peroxidase (Szili et al., 2017b).

#### 5.5.3 Cypridina luciferin and some other luciferins from biological sources

There are still other Chemiluminescence Assays used for detecting RONS in biological samples. Cypridina luciferin, a kind of Chemiluminescence Assay originally extracted from sea fireflies, could emit blue light in the presence of luciferase and oxygen. People subsequently developed analogs of cypridina luciferin to detect ROS (Yang et al., 2020).

### 5.6 Direct detection of CAP-Induced effects on tissues and cells

When assessing the depth of CAP effects on tissues, the cell cycle distribution, apoptosis, cell viability, and tissue antioxidant status can also be evaluated (Peng et al., 2024; Kos et al., 2017; Zhang et al., 2019; Szili et al., 2017b; Partecke et al., 2012; Arndt et al., 2018; Borchardt et al., 2017).

### 5.7 Some other methods

In addition to the above methods, several other techniques have been utilized to quantitatively assess CAP effects on tissues, as described below.

#### 5.7.1 Genetically engineered cells

These cells express compartment-specific ROS probes (e.g., the Hycer reporter and firefly luciferase gene) (Vandamme et al., 2010; Markvicheva et al., 2011; Gu et al., 2009; Bilan et al., 2013; Gast et al., 2022; Belousov et al., 2006).

#### 5.7.2 Raman microspectroscopy

Raman microspectroscopy can detect chemical bonds in living cells (e.g., lipids, proteins, nucleic acids) without exogenous fluorescent dyes or probes and is non-destructive (Smith et al., 2016; Wenzel et al., 2019; Ember et al., 2017). However, its shallow tissue penetration (typically <500 μm) limits deep-structure imaging and hence, its usefulness for assessing CAP effects in tissue depths (Ember et al., 2017; Imanbekova et al., 2022).

#### 5.7.3 Computer simulation methods

Some studies developed computer simulation methods that analyze the physical and chemical interaction mechanisms between plasma and liquids, primarily to model CAP-induced reactions in liquids (Chen et al., 2014; Zhang et al., 2019; Liu et al., 2016; Tian and Kushner, 2014).

## 6 Introduction of common tissue models and *ex vivo* and *in vivo* tissues

Because native tissues are compositionally and structurally complex and can limit probe penetration into cells, many studies used tissue models for experiments. Common tissue models include the following.

### 6.1 Liquid

Because biological tissues contain abundant water, aqueous solutions are the simplest tissue model. The reactive species in these solutions can be directly detected using methods such as ESR, colorimetry, and UV–visible spectroscopy. However, the penetration of CAP-generated reactive species in liquids is much greater than that in tissues.

### 6.2 Hydrogels

Hydrogels are also a relatively simple tissue model. Compared with aqueous solutions, hydrogels have physical properties that are more similar to native tissues, mainly in that: (a) they exhibit reduced fluidity; (b) they better mimic tissue water content and electrical properties; (c) some reagents or vesicles containing reagents can be homogeneously embedded in the hydrogel, enabling precise measurement of the depth of CAP effects in the hydrogel.

However, hydrogels still cannot adequately mimic native biological tissues because of the following factors. (a) Their structural strength remains lower than that of tough tissues such as skin. (b) They lack authentic cells, enzymes, blood flow, antioxidants, and complex microarchitecture. (c) They lack immune activity and metabolic functions. (d) They lack long-term stability and may suffer dehydration or aging. (e) They may contain air bubbles (Thulliez et al., 2021).

### 6.3 Tissue culture models

Tissue culture models offer both good physiological relevance and repeatability. 3D tissue co-culture models can accurately replicate the architecture of real tissues, cell–cell interactions, and cell–matrix signaling.

### 6.4 *Ex vivo* tissues

*Ex vivo* tissues retain a structural resemblance to *in vivo* tissues. However, disadvantages such as loss of cellular activity, tissue metabolism, immune function, and blood supply still remain. Furthermore, several detection probes do not penetrate well into cells as opposed to aqueous solutions and hydrogels, making the assays more complex and restrictive.

For real tissues (including both *in vivo* and *ex vivo* tissues), only certain detection methods are applicable owing to their structural complexity. The primary methods commonly used for assessing the penetration depth of CAP into living tissue include the use of (a) reagents that can penetrate cells without causing cytotoxicity and (b) certain indirect detection methods. For instance, by placing tissues on the surface of deionized water (or deionized water containing certain reagents), treating the tissue using CAP, and observing the results in the deionized water. If the presence of RONS in the deionized water can be demonstrated, it can be concluded that the CAP effect can penetrate the tissue thickness. However, as the tissue is in direct contact with the deionized water, this will lead to an increase in the tissue’s water content, thereby affecting accuracy to some degree.

Because of certain differences between aqueous solutions, hydrogels, *ex vivo* tissue, and *in vivo* tissue, the penetration depth of CAP varies accordingly. The subsequent section details the penetration depth of CAP for each distinct target material.

## 7 Summary of penetration depths

Table 2 contains the penetration depth of CAP in different types of tissue models and tissues from current studies.

**TABLE 2:** Summary of the penetration depth of CAP in different types of tissue models and tissues.

Subject	Plasma treatment	Detection target	Detection method	Penetration depth	References
Liquid					
Deionized water	O_2_ (1%)/He plasma jet, 5 min	ROS	KI-starch reagent	1 mm	Kawasaki et al. (2020)
Deionized water	Air surface microdischarge (SMD), t = 100 s	H_2_O_2_aq, O_3_aq	Indigo reagent, Amplex ® Red, Griess reagent, ESR	2 mm	Liu et al. (2015)
		NO_3_^−^aq, NO_2_^−^aq HNO_2_aq, N_2_Oaq		2–3 mm	
		OHaq, HO_2_aq, O_2_^−^aq		Degenerated *in situ*	
Normal saline	Air surface microdischarge (SMD), t = 100 s	HNO_3_/NO_3_^−^	Computer model	≈2 mm	Liu et al. (2016)
		N_2_O		1–2 mm	
		NO_2_		≈0.1 mm	
		HNO_2_/NO_2_^−^		≈0.04 mm	
		O_3_/H_2_O_2_		≈2 mm	
	t = 10 s	HClO/ClO^−^		≈0.3 mm	
		Cl_2_/ClNO_2_		0.1–0.2 mm	
	t = 100 s	HClO/HClO^−^		≈2 mm	
		Cl_2_/ClO_3_^−^		1 mm < c < 2 mm	
Deionized water	DBD, 3 discharge pulses and a 1 s afterglow	O_2_^−^, O_3_^−^, ONOO^−^, NO_3_^−^, H_2_O_2_, OH, HO_2_, O_3_	Computer model	>400 μm	Tian and Kushner (2014)
Hydrogels					
Gelatin tissue models	​	H_2_O_2_	2′, 7′-Dichlorodihydrofluorescein (DCFH), OPD/HRP	>1.5 mm	Szili et al. (2014)
Gelatin tissue models	Helium plasma jet, 15 s, 60 s, 300 s	Damaging effect of CAP on phospholipid vesicles	Vesicles encapsulating high concentrations of CF uniformly distributed throughout gelatin	>150 μm	Marshall et al. (2013)
Agarose tissue models	Helium plasma jet, t > 12.5 min	Effect of RONS in deionized water on optical absorbance	Place the agarose film over the deionized water, treat the agarose with CAP and then measure the absorbance in the deionized water	3.2 mm	Thulliez et al. (2021)
Gelatin tissue models	Air surface microdischarge (SMD), 5 min	NO_2_^−^, H_2_O_2_, O_3_	Place the gelatin film over the deionized water containing Griess reagent/Amplex® Red/Indigo carmine reagent, treat the gelatin with CAP	>1 mm	He et al. (2016)
Agarose tissue models	Helium plasma jet, 15 min	Effect of RONS in deionized water on optical absorbance	Place the agarose film over the deionized water, treat the agarose with CAP and then measure the absorbance in the deionized water	1.5 mm	Oh et al. (2015)
Agarose tissue models	Helium plasma jet, 15 min	Effect of RONS in deionized water on optical absorbance	Place the agarose film over the deionized water, treat the agarose with CAP and then measure the absorbance in the deionized water	1.5–5.8 mm	Szili et al. (2017b)
Gelatin tissue models	Helium linear-field and cross-field plasma jets, 5 min	NO_2_^−^	Place the gelatin film over the deionized water containing Griess reagent, treat the gelatin with CAP	1 mm	He et al. (2017)
Gelatin tissue models	Helium plasma jet, 1–10 min	ROS	Place the gelatin film over the deionized water containing DCFH reagent, treat the gelatin with CAP	>1 mm	Gaur et al. (2015)
Agarose tissue models	He or Ar plasma jet, 15 min	H_2_O_2_, NO_2_^−^, NO_3_^−^, O_2_	Place the agarose film over the deionized water, treat the agarose with CAP and then measure the absorbance in the deionized water	>5 mm	Oh et al. (2016)
Agarose tissue models	Helium plasma jet, 5 min	H_2_O_2_, NO_2_^−^, NO_3_^−^, O_2_	Place the agarose film over the deionized water, treat the agarose with CAP and then measure the absorbance in the deionized water	>1.5 mm	Oh et al. (2015)
Agarose tissue models	Helium plasma jet, 15 min	H_2_O_2_, NO_2_^−^, NO_3_^−^, O_2_	Place the agarose film over the deionized water, treat the agarose with CAP and then measure the absorbance in the deionized water	>4 mm	Oh et al. (2015)
Agarose tissue models	Helium plasma jet, 30 min	H_2_O_2_, NO_2_^−^, NO_3_^−^	Place the agarose film over the deionized water, treat the agarose with CAP and then measure the absorbance in the deionized water	>3.2 mm	Szili et al. (2015)
Gelatin tissue models	He+0.5%O_2_+10 ppm O_3_ plasma jet, 4–5 min	ROS	KI-starch reagent	≈470 μm	Liu et al. (2018b)
Agarose tissue models	He or O_2_ (1%)/He plasma jet, 5–7 min	ROS	KI-starch reagent	>1 mm	Kawasaki et al. (2016)
Agarose tissue models	O_2_ (1%)/He plasma jet, 6 min	ROS	KI-starch reagent	2 mm	Kawasaki et al. (2019)
Agarose tissue models	Argon plasma jet	ROS	KI-starch reagent	6 mm (6 min) 8 mm (36 min) 11 mm (66 min)	Ghimire et al. (2019)
Gelatin tissue models	Helium plasma jet,the air flow is 0.5 slpm (15–60 s) or 0.05 slpm (10–20 min)	DNA-strand breaks, vesicle poration/rupture, Measurement of H_2_O_2_ concentration	Molecular beacon, Vesicles encapsulating high concentrations of CF, OPD/HRP	>2 mm	Szili et al. (2017a)
Highly hydrated biofilms and plasma-tissue interaction models	Low-power He-O_2_ plasma	ROS	Model framework	H_2_O_2_, O_2_^−^: 1–1.2 mm HO_2_: 20–250 μm O_3_: 5–40 μm	Chen et al. (2014)
Agarose tissue models	FE-DBD, 30–120 s	H_2_O_2_	Amplex ® Red	2–5 mm (also affected by mass fraction of agarose)	Dobrynin et al. (2012)
Tissue culture models					
*In vitro* cultured human pancreatic adenocarcinoma	t = 10, 20 s	Cell viability and apoptosis *in vitro*	TREG-detection kit, Annexin-V-FITC/DAPI-Assay, immunohistochemistry analysis	10 s: 36.8 ± 14.2 μm。 20 s: 48.8 ± 12.3 μm。	Partecke et al. (2012)
*In vitro* 3D-cultured human A549 lung carcinoma	t = 5 min (indirect treatment)	Cell viability	Cell-Titer-Glo® luminescent cell viability assay kit	130 μm	Zhang et al. (2019)
		RONS	Amplex® Red,Griess reagent	>175 μm (penetrate into the center of the 3D cancer spheroids)	
	t = 1 min	Long-lasting species (H_2_O_2,_ NO_2_^−^ and NO_3_^−^)	Model framework	>1 mm	
*In vitro* cultured cervical cancer (CC) cell line SiHa	Ar plasma jet, 5–120 s	Cell proliferation and associated molecular and biochemical changes of single cells	Cell counting and Raman microspectroscopy	270 μm	Wenzel et al. (2019)
*Ex vivo* tissues					
Pig skin connected to a 1 mm layer of sub-cutaneous fat	Helium plasma jet, t = 15 min	RONS	DCFH-DA	>1 mm	Szili et al. (2017b)
Pig muscle tissues	10 min	H_2_O_2_	Place the pig muscle tissues over the deionized water, treat muscle tissues with CAP.	750 μm	Nie et al. (2018b)
	5–15 min	O_3_		<500 μm	
	10–15 min	NO_2_^−^, NO_3_^−^		1.25 mm	
	15 min	Total RONS		1.25 mm	
*Ex vivo* rat skin tissue	FE-DBD, 30–120 s	H_2_O_2_	Amplex® UltraRed is injected subcutaneously into the rat tissue	2–4 mm	Dobrynin et al. (2012)
Skinless chicken breast tissue	FE-DBD, 60–120 s	pH	Fluorescein (Sigma Aldrich) is injected into tissue	Up to 4.5–5 mm	
	FE-DBD, 30–120 s	H_2_O_2_	Amplex ® Red	1.5–3.5 mm	
Mouse skin punctured with (or without) microneedles	Argon plasma jet (kINPen09), 10 min	RONS	Place the mouse skin over the deionized water, treat the skin with CAP (directly or through PAW) and then measure the absorbance in the deionized water	<0.75 mm (even when using the microneedles)	Liu et al. (2018a)
	Plasma activated water (PAW)			>0.75 mm (deeper than direct treatment)	
Pig muscle tissue	He mixed with 0.5% O2, 5–20 min	H_2_O_2_, NO_2_^−^ and NO_3_^−^, pH	Place the pig muscle over some liquids with hydrogen peroxide assay or Griess reagents, treat the muscle with CAP, (six different types of liquids: double-distilled water (DDW), 1% phosphate-buffered saline (PBS), 0.9% NaCl, 5% glucose, 2% serum, 10% serum solution)	500–2000 μm (affected by the type of liquid)	Nie et al. (2018a)
Hair follicles on pig ears	kINPen09, 30 min	CAP can induce chlorophyll to fluoresce	Chlorophyll dye-containing particle solution	300–400 μm	Lademann et al. (2011)
Living tissues					
Cancer cell apoptosis within an 2.8 ± 0.5 mm thick tumor, grown on the back of a live rodent	Helium plasma jet, 15 min	Apoptosis	TUNEL signals	2.8 mm	Szili et al. (2017b)
		ROS	Intraperitoneal injection of Luminol solution	ROS spread throughout the body	
Tumor xenograft model (Calu-1 cells) in nude mice	Helium plasma jet, 20 days (15 min every 2 days)	Oxidative stress and cellular damage	4-HNE and TUNEL signals	<500 μm	Peng et al. (2024)
U87-Luc glioma tumor (a human malignant glioma cell line) cultured subcutaneously in Balb/c nude female mice	DBD, gas mixtures of air with argon, 20 min, five consecutive days	Degree of tumor reduction and tumor activity	Cell line is stably transfected with firefly luciferase gene	CAP can penetrate deep into the subcutaneous tumor tissue	Vandamme et al. (2010)
Mice skin wounds	MicroPlaSter ß1, 2 min, 10 days long	Vascular density	quantitative RT-PCR,mRNA expression of CD31 and FGF-2	≈65 μm	(Arndt et al., 2018; Privat-Maldonado et al., 2019) (90 provides the images, whereas 111 measures the effective depth.)
Mice skin tissues	Helium plasma jet, 1–5 min	skin damage	FITC labelled dextran	≈50 μm	(Kos et al., 2017; Privat-Maldonado et al., 2019) (59 provides the images, whereas 111 measures the effective depth.)
Dorsal skin of the forearm (10 Healthy volunteers)	90, 180, 270 s	Local microcirculation within 1–2 mm depth of the skin	Noninvasive optical system Oxygen-to-see (O2C)	1–2 mm	Borchardt et al. (2017)
Forearm skin (seven healthy volunteers)	kINPen09, argon plasma jet, 3 s and measurements were completed within 5 min after CAP treatment	Valid marker substances for the complete antioxidative network of the human organism	Raman microspectroscopy, the carotenoids in the human skin	10 μm	Fluhr et al. (2012)

## 8 Current clinical trials on CAP

These clinical trials also confirm the efficacy of CAP in anti-inflammatory, wound-healing, and anti-tumor applications. Table 3 contains some clinical traits of CAP. When using higher doses of CAP, typically the short-lived reactive species, the electric field strength, and UV intensity all tend to decay during the penetration process. And in deeper tissues, short-lived reactive particles are rare, while long-lived reactive species (such as H_2_O_2_, NO_2_^−^) and certain liquid-phase reaction products can reach deeper layers through diffusion, convection, or via appendages like hair follicles or sweat glands. Taking skin as an example, when CAP acts on the skin, it often produces a strong disinfecting effect and regulatory effects on skin barrier function in the epidermal layer, High concentrations of reactive oxygen and nitrogen species (RONS, such as ·OH, O_2_·^-^, H_2_O_2_, ONOO^−^), UV, and transient electric fields in the epidermis can cause oxidation of lipids, proteins, DNA, and other substances, promoting cell death or apoptosis. Simultaneously, disruption of cell membrane lipids leads to membrane rupture, which reduces the barrier function of the epidermis, facilitating deeper penetration of these active ingredients. This physical, non-specific killing mechanism makes it difficult for microorganisms to develop resistance, offering a new strategy for treating infections caused by drug-resistant bacteria. At deeper tissue levels, CAP primarily functions by improving microcirculation and promoting cell proliferation. However, the number of existing clinical studies is too small, and no clinical research has yet observed the systemic effects of CAP. In a live experiment on mice, elevated ROS levels were detected in other parts of the mouse’s body following local treatment with CAP (Szili et al., 2017b). Nevertheless, this has not yet been investigated in human *in vivo* studies. Moreover, due to variations in the CAP parameters and application methods used across different clinical trials, the results obtained also differ. There is an urgent need to standardize the operational parameters and application methods of CAP to ensure treatment safety and promote more precise therapy.

**TABLE 3:** Clinical trials of CAP. The trials that included quantitative analysis of the depth of action of CAP are described in Table 2. The following are clinical trials that did not perform quantitative analysis of the depth of action of CAP.

Subject	Plasma treatment	Effect	References
Patients with pyoderma gangrenosum (PG)	12 weeks, with two direct-CAP treatments per week	Statistically significant reduction in fibrin coatings	Gewis et al. (2025)
Patients with venous leg ulcers (VLUs)	Direct-CAP once or twice a week, for 12 weeks or until healing	Higher percentage of wounds healed	Bakker et al. (2025)
Patients with diabetic foot ulcers	8 applications of argon plasma	Significantly improved the healing process	Stratmann et al. (2020)
Patients with diabetic foot	About 14 days	Increased levels of FGF-2 and VEGF-A. increased levels of tumour necrosis factor-alpha, interleukins 1α and 8. The total protein amounts and the total protein were not significantly elevated	Hiller et al. (2022)
Patients with diabetic foot ulcers	3 times per week, 3 weeks, helium plasma	CAP accelerates wound closure and decreases bacterial load	Mirpour et al. (2020)
Patients with diabetic foot ulcers	6-week treatment, 2 times per week, and an 8-week follow-up, helium plasma jet, at a dose of 1 min/cm^2^ of wound size	The amount of exudate, wound grading and the ulcer size are all decreased	Samsavar et al. (2021)
Patients with chronic infected wounds	2 min per time, once a day	Highly significant reduction in bacterial load	Isbary et al. (2012)
Patients with therapy-refractory chronic wounds	1 or 3 times per week, the maximum treatment duration was set at 12 weeks	Wound area and bacterial load decreased significantly, pain reduced significantly. And once weekly treatment with CAP were not inferior to those obtained when CAP treatment was three times a week	Moelleken et al. (2020)
Intact skin of human volunteers that was contaminated with *P. aeruginosa*	A flexible DBD plasma pad, 3 times for 20 s with plasma on separated by 2 intervals for 10 s with plasma off	The mean log CFU reduction was 2.9 and was not significantly affected by plasma power setting. Transient pain, increased skin temperature, and erythema may be observed	Boekema et al. (2021)
Patients with pruritus	Argon plasma jet, 2 min per day	No result in higher pruritus reduction than that in the treatment with argon gas only	Heinlin et al. (2013a)
Patients with atopic dermatitis (AD)	Argon plasma jet, 5mins per time, once a week, last for 3 weeks	CAP has the potential to effectively improve the severity of mild and moderate AD	Kim et al. (2021)
Forty patients with skin graft donor sites on the upper leg	Argon plasma jet, 2 min a time and were conducted daily except for the weekend	Considerable positive effects could be observed with regard to improved reepithelialization, significantly fewer fibrin layers, and blood crusts, without any influence on wound surroundings	Heinlin et al. (2013b)
Patients with chronic wounds	1 min per time, three times during the first week, twice during the second and third weeks, and once weekly starting from the fourth week	CAP demonstrates excellent efficacy in promoting wound healing, reducing pain, and minimizing exudate	Strohal et al. (2025)
*Patients with Malassezia* folliculitis	3 min a time, once a day, last for 2 weeks	CAP demonstrated significant antifungal activity against *Malassezia* yeasts	Wang et al. (2024b)
Patients with split skin graft donor sites	Three times daily for 90 s each session, for 7 consecutive days	The CAP wound dressing was superior to the control (*p* < 0.001) in the improvement of 3 wound parameters, that is, deep tissue oxygen saturation, hemoglobin distribution, and tissue water distribution	van Welzen et al. (2021)
Patients with keloids	BIOplasma® system (DBD), twice a week, a total of 5 times, 5–15 min per time	The color, pigmentation, redness, texture, and volume were all improved after the treatment	Suwanchinda and Nararatwanchai (2022a)
Patients with rosacea	90 s per time, once a day, for 6 weeks, DBD device PlasmaDerm® Flex	CAP is a promising new treatment of rosacea	Hofmeyer et al. (2023)
Patients with striae distensae	Once every 2 weeks, for a total of five sessions	Adverse effects included small scabs, shallow wounds, and rash	Suwanchinda and Nararatwanchai (2022b)
Patients with symmetric melasma	Both sides were treated with topical hydroquinone 4% every night, and one side of the face was randomly selected for eight weekly treatment sessions with two passes of non-thermal plasma	Combined CAP therapy yields better results	Yousefi et al. (2023)
Healthy female hand skin	Nitrogen plasma jet, once a week, for a total of 8 sessions	Significant improvement in wrinkles and dyschromia, and boost skin hydration	Hadian et al. (2022)
Patients with locally advanced (pT4) squamous cell carcinoma of the oropharynx suffering from open infected ulcerations	Plasma jet (kINPen MED), 3 times a week, followed by an intermittence of 1 week	Demonstrate a moderate amount of apoptotic tumor cells and a desmoplastic reaction of the connective tissue	Metelmann et al. (2018)
Patients with actinic keratoses	SteriPlas, Adtec®, twice weekly for 3 min	CAP treatment showed significantly better effectiveness over diclofenac in reducing the lesion count	Koch et al. (2020)
Patients with stage IV or recurrent solid tumors underwent surgical resection combined with intra-operative CAP treatment	Patients were treated with CHCP intra-operatively at the surgical margin site after macroscopic tumor resection	Combining CAP with surgical resection can reduce the recurrence rate of solid tumors	Canady et al. (2023)
Women Positive for Cervical Intraepithelial Neoplasia	Helium plasma	Demonstrated a Significant therapeutic effect	Marzi et al. (2022)
Patients with cervical intraepithelial neoplasia	VIO3/APC3 and 3.2 mm APC probes (preciseAPC setting, effect 1) at a rate of 30 s/cm^2^	Achieve full histological remission in 86.2% and improvement of cytological findings in 52.7% of patients	Weiss et al. (2023)
Healthy skin	90 s, 180 s, and 270 s	Significant increases in microcirculation were observed	Borchardt et al. (2017)
Healthy skin	PlasmaDerm® FLEX9060 (DBD), 3 times	Repeated application results in greater increases in oxygen saturation, significantly prolonged duration, and enhanced peak blood flow	Kisch et al. (2016)
Patients with denture stomatitis	kINPen MED, once a week, 6 weeks	CAP can significantly accelerate the fading of erythema, but it did not significantly reduce *Candida* load	Preissner et al. (2016)
Patients with periodontitis	CAP was placed in the pocket after the Scaling and root planing (SRP), each tooth for 2.5 min	CAP adjunctive therapy can reduce the recurrence rate of periodontal disease	Küçük et al. (2020)
Patients who required implant placement in the maxillary arch	Using CAP for healing abutments	Showed a better effect on the peri-implant soft tissues by reducing the inflammatory reaction, promoting collagen fiber formation, higher fibroblast-like cell attachment, and upregulating E-cadherin expression	Yossri et al. (2025)
Complete skin (fingertips)	The atmospheric pressure plasma (pulsed and non-pulsed) jet (APPJ), kINPen 09	All plasma treatments were well-tolerated and did not damage the skin barrier nor cause skin dryness	Daeschlein et al. (2012)

## 9 Conclusion

Numerous studies have measured the penetration depth of CAP into tissue models or tissues, and the results obtained vary considerably. Many factors influence the effect of CAP on tissue models or tissues, such as the use of CAP generators with different parameters, different tissue types, and varying detection methods. By adjusting these variables, the scope of CAP’s action can be modulated to achieve the desired histological level, thereby advancing research into CAP’s mechanism of action on tissue. This approach also guides the selection of indications and the adjustment of CAP usage parameters, further enhancing the precision and safety of CAP treatment. We look forward to further standardization of CAP treatment to advance its broader clinical application.
